# Intermittent Massage as a Therapeutic Option for Compartment Syndrome after Embolectomy of the Lower Limbs

**DOI:** 10.1155/2018/2679358

**Published:** 2018-05-02

**Authors:** José Maria Pereira de Godoy, Maria de Fátima Guerreiro Godoy

**Affiliations:** ^1^Cardiology and Cardiovascular Surgery Department, Medical School in São José do Rio Preto (FAMERP), São José do Rio Preto, SP, Brazil; ^2^Clínica Godoy, São José do Rio Preto, SP, Brazil; ^3^Medical School in São José do Rio Preto (FAMERP), São José do Rio Preto, SP, Brazil; ^4^Research Group of the Clínica Godoy, São José do Rio Preto, SP, Brazil

## Abstract

The case of a 54-year-old cardiac patient is reported, who was admitted to hospital with a complaint of sudden pain in the legs associated with edema, paresthesia, and coldness. Arterial embolism of the lower limbs was diagnosed and the patient was submitted to bilateral embolectomy. The patient evolved with a burning sensation, hypersensitivity in the right leg, swelling, and difficulty bending and stretching the sole of the foot and the knee. A physical examination detected edema and increased tension in the anterior, lateral, and posterior compartments. Treatment using intermittent massage of the leg during the evaluation of the patient was chosen in an attempt to stimulate lymphatic and venous drainage. After a few minutes of stimulation, there was significant improvement in the pain and edema. In 40 minutes, there was total reduction of the pain in the posterior and lateral compartments and improvement of over 50% in the anterior compartment. After this, the patient started to bend the knee without pain and bend the sole of the foot with slight pain. On the following day, the patient was walking around the hospital ward without difficulty. It seems that intermittent massage is a therapeutic option in selected cases of compartment syndrome.

## 1. Introduction

Acute compartment syndrome is a devastating complication after trauma of the extremities [1]. It is defined as an interstitial pressure increase in the osteoarthritis space involving muscles and vessels in such a way that the microcirculation is compressed and tissue perfusion is impaired [2].

The diagnosis of compartment syndrome of the lower limbs is clinical in priority; however measurements of the pressure can be carried out to confirm the diagnosis and plan treatment [3]. Classic treatment involves surgical decompression with an immediate fasciotomy considered essential to prevent disability and loss of the extremity [4].

The main causes of acute arterial insufficiency are thrombosis and embolism; it is possible that complications occur during their respective treatments including compartmental syndrome and reperfusion syndrome [5]. Surgical decompression is the main technique used to rapidly reduce the pressure of the compartment. The use of techniques to stimulate lymphatic and venous return quickly has not been reported in the literature.

The aim of this study is to report on a new therapeutic option in compartment syndrome, the intermittent massage of involved compartments.

## 2. Case Report

The case of a 54-year-old male patient is reported. The patient, an ex-smoker, with systemic arterial hypertension (SAH) and heart failure (Chagasic cardiomyopathy with implanted pacemaker) had been submitted to coronary angioplasty. On being admitted to the emergency room of the University Hospital de Base in São José do Rio Preto, the patient reported he had pain in the right lower limb associated with edema, paresthesia, and coldness that had started suddenly. He also complained of pain in the left leg but said the pain was less intense. A duplex ultrasound of the iliac arteries showed total occlusion by an intraluminal thrombus on the right side with the superficial femoral and popliteal arteries occluded along their complete length. The popliteal artery of the left leg was also distally occluded (bifurcation). Bilateral embolectomy was performed without complications.

On the following day, he complained of “burning” pain and said that even the blanket on his legs bothered him. He had difficulty bending and straightening his knee and the sole of his foot. On physical examination, the patient had edema of the lateral and posterior compartments, and a lot of pain on compression, especially of the anterior compartment that was highly sensitive to slight pressure.

The chosen conduct was intermittent manual compression (massage) of the musculature of the compartments with the objective of stimulating lymphatic and venous return during the physical examination. The posterior compartment had a 100% improvement in the pain after 20 minutes of massage, the lateral compartment improved the pain completely after around 40 minutes, and the anterior compartment improved the pain by about 70% within one hour. With this improvement, the patient spontaneously bent his knee (without pain) and the sole of his foot (with a little pain in the anterior compartment). The patient was asked to evaluate his pain using an analog scale with the worst imaginary being 10 and no pain at all being 0. This was the only therapy used to reduce the edema, because the patient does not tolerate cardiac overload. The following day, the patient was walking around the ward without difficulties.

## 3. Discussion

This study brings a new therapeutic option for reperfusion-induced compartment syndrome. The pathophysiology of this syndrome involves a pressure increase in the compartment caused by edema. Thus, the use of maneuvers to enhance venous and lymphatic drainage is a therapeutic option in selected cases. Manual compression of the muscle was carried out; this is different from superficial lymph drainage because it increases the pressure in the vessels and cell interstice. This approach has not been described in the literature.

Intermittent compression drains both the superficial and deep systems, and in a few minutes there was a visible reduction in the edema and clinical improvement. The use of intermittent compression allows the vessel to collapse thereby emptying it and assisting the interstitial fluid to reach the micro- and macrocirculation.

It was decided to drain the least sore compartments first (in ascending order): starting with the posterior, then the lateral, and finally the anterior. The compression used was well tolerated by the patient and pain was constantly monitored. Initially, slight compression was employed but as the pain improved, the intensity of the compression was increased.

The posterior compartment improved fastest during treatment; the patient was constantly asked about how much pain he felt based on an analog pain scale. The strategy was to begin with the posterior compartment (less painful); however very gentle massage was performed on the other two compartments simultaneously. As the pain improved, the massage maneuvers became more frequent.

This is the fifth case that this approach has been used by the researcher, but only one other, in a newborn baby (in the process of publication), was documented. And so this is the second well-documented case suggesting that carefully evaluated patients may benefit from this treatment option. One advantage of this conduct is that the feasibility of using the approach is tested within a few minutes. If the patient says that he feels less pain within minutes of starting the massage, then it is possible to continue with the procedure. The other option, decompression fasciotomy, is surgery.

The great advantage of this conduct is the speed at which the symptoms improve; as it is a clinical therapy, the risks of surgery and the long postoperative period are avoided. The patient was discharged the following day, walking without pain with complete normalization of the leg being achieved within a few days.

The history and physical examination left no doubt as to the diagnosis of compartment syndrome and the therapeutic test was initiated immediately upon diagnosis. The patient had difficulty using a blanket over the leg due to hypersensitivity. Within a short time (less than 10 minutes), an improvement was already reported by the patient and was observed by the professional who could note a reduction in the swelling in the regions where limb compression was being performed.

Intermittent compression maneuvers involving subcutaneous tissue and muscles increase the pressure in the compartment thereby increasing the pressure differential in the lymphatics, veins and interstitial space, and favoring drainage. This maneuver is not specifically lymph drainage which uses pressures of less than 40 mmHg; it is more like massage because it involves compression of the muscles.

Compartment syndrome causes a lot of doubts in respect to the diagnosis with the possibility that opinions change during the evaluation of the patient. When there is enough time, a direct measurement of the compartment pressure is recommended, but an alternative when this is difficult is to perform a diagnostic mini-fasciotomy. On detecting high blood pressure in a compartment, a complete fasciotomy should be performed. On the other hand, if there is no high blood pressure in the compartment, the diagnostic incision should be sutured [6]. In case of hematoma, the pathophysiology is another and fasciotomy is obligatory. In the trauma, the deep comparison is more affected where the massage will not be effective. Therefore, each patient should be evaluated in person.

## 4. Conclusion

Intermittent massage may be a therapeutic option in selected cases of compartment syndrome.

## References

[1] Branco B. C., Inaba K., Barmparas G. (2011). Incidence and predictors for the need for fasciotomy after extremity trauma: A 10-year review in a mature level i trauma centre.

[2] Kalns J., Cox J., Baskin J., Santos A., Odland R., Fecura S. (2011). Threshold model for extremity compartment syndrome in swine.

[3] Masquelet A.-C. (2010). Acute compartment syndrome of the leg: Pressure measurement and fasciotomy.

[4] Wittstein J., Moorman C. T., Levin L. S. (2010). Endoscopic compartment release for chronic exertional compartment syndrome: Surgical technique and results.

[5] Locatelli E. C., Pelizzari S., Scapini K. B. (2009). Exercícios físicos na doença arterial obstrutiva periférica.

[6] Godoy J. M. P., Godoy M. F., Silva A. A. M., Reis L. F. (1998). Minifasciotomia diagnóstica na síndrome compartimental.

